# Gut microbiota derived from fecal microbiota transplantation enhances body weight of Mimas squabs

**DOI:** 10.5713/ab.23.0475

**Published:** 2024-04-01

**Authors:** Jing Ren, Yumei Li, Hongyu Ni, Yan Zhang, Puze Zhao, Qingxing Xiao, Xiaoqing Hong, Ziyi Zhang, Yijing Yin, Xiaohui Li, Yonghong Zhang, Yuwei Yang

**Affiliations:** 1 College of Animal Science, Jilin University, Changchun 130062, China; 2 College of Animal Science and Technology, Jilin Agricultural Science and Technology University, Jilin 132109, China; 3 Center of Animal Experiment, College of Basic Medical Sciences, Jilin University, Changchun 130021, China

**Keywords:** Body Weight, Fecal Microbiota Transplantation, Intestinal Microbial Diversity, Pigeons, 16S rRNA Sequencing

## Abstract

**Objective:**

Compared to Mimas pigeons, Shiqi pigeons exhibit greater tolerance to coarse feeding because of their abundant gut microbiota. Here, to investigate the potential of utilizing intestinal flora derived from Shiqi pigeons, the intestinal flora and body indices of Mimas squabs were evaluated after fecal microbiota transplantation (FMT) from donors.

**Methods:**

A total of 90 one-day-old squabs were randomly divided into the control group (CON), the low-concentration group (LC) and the high-concentration group (HC): gavaged with 200 μL of bacterial solution at concentrations of 0, 0.1, and 0.2 g/15 mL, respectively.

**Results:**

The results suggested that FMT improved the body weight of Mimas squabs in the HC and LC groups (p<0.01), and 0.1 g/15 mL was the optimal dose during FMT. After 16S rRNA sequencing was performed, compared to those in the CON group, the abundance levels of microflora, especially *Lactobacillus*, *Muribaculaceae*, and *Megasphaera* (p<0.05), in the FMT-treated groups were markedly greater. Random forest analysis indicated that the main functions of key microbes involve pathways associated with metabolism, further illustrating their important role in the host body.

**Conclusion:**

FMT has been determined to be a viable method for augmenting the weight and intestinal microbiota of squabs, representing a unique avenue for enhancing the economic feasibility of squab breeding.

## INTRODUCTION

The intestinal microbiota is a complex ecosystem that is intricately associated with the growth performance and health status of animals [1]. In recent years, this topic has received considerable attention. The manipulation of the gut microbiota represents a novel strategy for improving the growth rate, feed utilization, and immune performance of livestock and poultry. Fecal microbiota transplantation (FMT) has the potential to facilitate the transfer of gut microbiota across different species, and may also influence the early colonization of the gut microbiota in young poultry. Research has demonstrated that transplanting cecal microbiota from adult chickens to chicks can enhance their immunity against *Salmonella* infection and decrease mortality rates [2]. Similarly, the introduction of *Lactobacillus* during the initial week after chick birth can promote the colonization of advantageous bacteria and enhance feed conversion efficiency [3]. Additionally, the provision of beneficial bacteria (*Lactobacillus salivarius* and *Lactobacillus agilis*) to hens has been observed to significantly enhance egg laying rates [4]. Currently, research on FMT has primarily focused on common type of poultry [2–4], with no available literature on FMT investigations pertaining to emerging breeding varieties, such as pigeons.

Poultry meat is a crucial source of dietary protein in nearly every country worldwide [5,6]. The pigeon, a species of avian that is specific, is a sought-after delicacy for its meat, which is high in protein, has a tender texture, and has a low-fat content. It has been estimated that the digestion rate of pigeon meat is as high as 97% [7,8]. In light of the ongoing improvement in the comprehensive production capacity of animal husbandry, there has been a growing demand for such high-quality poultry products [9]. The squab possesses unique physiological traits that enable it to reach adult size within a month [10], but impede its ability to independently leave the nest and forage upon hatching [11]. Fledglings rely on pigeon milk secreted by their parents, resulting in a comparatively low feeding survival rate for squab, thereby constraining the enhancement of breeding efficiency for this species [11,12]. The Mimas meat pigeon is a breed of meat pigeon that is a marketable product due to its exceptional egg-laying ability, proficient lactation, and well-developed edible musculature. In contrast to Mimas pigeons, Shiqi pigeons, a native breed in China, exhibit heightened resistance to diseases and stress, displaying a diverse range of feed utilization and heightened adaptability to environmental conditions [13,14]. An effective technique is expected to be employed to improve the environmental adaptability of Mimas pigeons.

Several studies have demonstrated that the isolation of *Bacillus velezensis* from pigeon feces can significantly enhance the resistance of pigeons to pigeon circovirus invasion [15]. The administration of *Bifidobacterium* to domestic pigeons has been determined to have a significant effect on weight gain. Moreover, it has been shown to augment the bursa of Fabricius index and elevate the levels of serum immunoglobulin M [16]. However, reports on the enhancement of pigeon production performance by the gut microbiota are rare. Consequently, we investigated whether fecal bacteria transplantation can be used to take advantage of the intestinal flora resources of Shiqi pigeons to effectively enhance the colonization of the intestinal flora in Mimas squabs and enhance their growth performance. The body weight and gut microbiota of Mimas squabs were measured in this FMT experiment, hoping to provide innovative perspectives for identifying a new approach for the high-quality development of Mimas squab breeding.

## MATERIALS AND METHODS

### Animal care

The present experiment was reviewed and approved by the College of Animal Science of Jilin University Ethics Committee (SY202306051).

### Prepared bacterial solution

A total of fourteen healthy feral Shiqi pigeons not treated with antibiotics within three months were used as fecal donors. All the pigeons in this study were provided by Jilin Sheng Chengcheng Agricultural Development Co., Ltd (Liaoyuan, Jilin, China). All the Shiqi pigeons were slaughtered using exsanguination. The contents of ileum were collected from each Shiqi pigeon and thoroughly mixed in sterile tubes before using; 0.1 and 0.2 g of fresh ileal contents samples were mixed with 15 mL of cryoprotectant solution (30% glycerol be formulated in 0.9% NaCl), and strained into sterile disposable pipettes to produce low-concentration and high-concentration bacterial solutions, respectively, that were free of impurities. The bacterial solution preparation process was controlled within 1 h, after which the samples were sub packed and quickly transferred to an ultralow temperature freezer at −80°C for storage until use.

### Animals, diet, and experimental design

Meat pigeon breeding adopted a “2+3” production mode, as three squabs were raised by a pair of pigeons (one female and one male) per cage. A total of 90 at 1-day-old Mimas squabs (the day of hatching) were randomly assigned to three treatment groups, with 3 replicates of 10 each. The above-mentioned bacterial solution was thawed gently on ice just prior to use in the experiments. The bacterial solution was collected into a sterile 1 mL syringe with a straight 12-gauge needle. The solution was gavage-fed to squabs at 10:00 AM daily after they were fed by parental pigeons. At 2 days of age, 4 days of age and 6 days of age, the HC group (high-concentration group) and the LC group (low-concentration group) were gavaged with 200 μL of high-concentration and low-concentration bacterial solution, respectively. The control group was fed an equivalent amount of 30% glycerol solution (prepared with 0.9% NaCl) by gavage. The Mimas meat pigeons were reared under identical environmental conditions. The squabs in the three groups were fed with the same basic diet, which included a pellet diet and raw grain according to the pigeons’ feeding habits and actual production conditions (Table 1). The basic diet was formulated according to the NRC (1994) recommendations. The parental pigeons were allowed unrestricted access to food and water and were solely responsible for feeding the squabs without any additional artificial supplementation during the trial. The Mimas squabs received no other medications during the feeding regimen, except for routine immunization procedures. The feeding experiment was conducted over a period of 28 days.

### Performance parameter measurements and sample collection

All birds were individually weighed at 1 day of age and subsequently on a weekly basis to estimate average daily gain (ADG) throughout the trial. The weekly ADG was calculated as follows:


[Body weight on specific week (g)-initial body weight (g)]/7

On the final day of the experiment, 10 of the 30 squabs in each group whose body weight was close to the average body weight were selected for slaughter by decapitation. The ileal digesta contents of each squab were collected separately with a sterile instrument in sterile tubes and stored at −80°C for gut microbiota analysis.

### DNA extraction and ileal microbiota analysis

A total of ten ileal samples from each group were randomly selected for sequencing. Sequencing was performed by Shanghai Applied Protein Technology Co., Ltd (Shanghai, China). Total DNA was extracted from the intestinal contents using Magnetic Soil and Stool DNA Kit (TIANGEN, Beijing, China) following the manufacturer’s instructions. Extracted genomic DNA was detected via 1% agarose gel electrophoresis. Polymerase chain reaction (PCR) amplification of gene V3–V4 region was conducted using primers 341F-(CCTAYGGGRBGCASCAG) and 806R-(GGACTAC NNGGGTATCTAAT) by Trans Start Fast pfu DNA Polymerase using Phusion High-Fidelity PCR Master Mix (New England Biolabs, Beverly, MA, USA). The samples were subsequently purified using a Qiagen Gel Extraction Kit (Qiagen, Dusseldorf, Germany) beads purification protocol. Constructing 250 bp paired-end raw sequencing data and sequenced using the Illumina NovaSeq 6000 platform (Illumina Inc, San Diego, CA, USA). The generated fastq format files were ultimately converted to fasta format files through Trimmomatic (version 0.36), Pear (version 0.9.6), Flash (version 1.20) and Vsearch (version 2.7.1). QIIME2 (version 2020.9) was used to analyze the obtained raw sequences, and the trimmed sequences were quality-checked and denoised for amplicon sequence variants (ASVs) by DADA2. Alpha diversity indices (goods_coverage, Chao 1, Pielou, Shannon and Simpson indices) and beta diversity were calculated using QIIME2 software, and UniFrac distance matrices were used to visualize the relationships among the samples via principal coordinates analysis (PCoA). The analysis of similarities (ANOSIM) was carried out with the R package vegan. The taxonomy of each ASV sequence was analyzed by an RDP classifier algorithm. Taxonomic classification was performed using the QIIME2 feature-classifier and the data were visualized with the R package ggplot2. Statistical differences in bacteria and Spearman correlation coefficients were analyzed via GeneCloud (https://www.genescloud). Functional prediction of bacteria was conducted using PICRUSt (Phylogenetic Investigation of Communities by Reconstruction of Unobserved States) (https://picrust.github.com/picrust) and the results were compared via PCoA and ANOSIM. The predicted functional differences were tested by metagenomeSeq (R package) and Random_Forest. Spearman correlation analysis was performed with GenesCloud (https://www.genescloud).

### Statistics and analysis

One-way analysis of variance was used to analyze the weight and ADG data using SPSS software version 27.0 (SPSS, Inc., Chicago, IL, USA), and the acquired data were subsequently plotted with OriginPro software version 2023b (OriginLab Corporation, Northampton, MA, USA). All the weight data are expressed as the means±standard error. Statistical differences in bacteria were analyzed by the Mann-Whitney test with the GraphPad Prism statistical package. *, **, and *** denote significant differences at p<0.05, 0.01, and 0.001, respectively.

### Accession numbers

The high throughput sequencing datasets presented in this study are openly available in the NCBI’s Sequence Read Archive (SRA, https://www.ncbi.nlm.nih.gov/sra) with accession number PRJNA987322.

## RESULTS

### Growth performance of squabs

**Figure 1:**
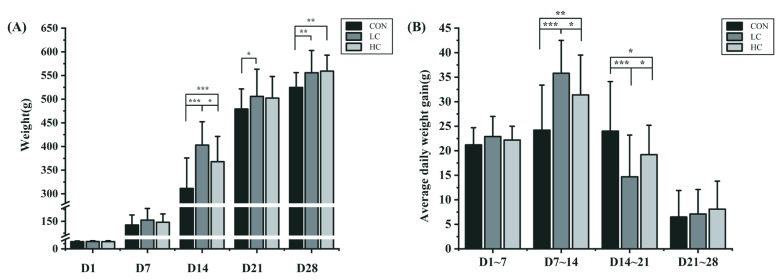
The weight data of the Mimas squabs. (A) The weekly body weight of the HC group (high-concentration group), the LC group (low-concentration group) and the CON group (control group). (B) The average daily gain (ADG) of each group. The day of birth was defined as D1 (1-day-old). Each bar represents the mean and standard error. Indicates a difference at * p<0.05, ** at p<0.01, *** at p<0.001.

The body weight of the squabs was measured every week (Figure 1A). There was no significant difference in the body weight of the squabs among the three groups (p>0.05) before 14 days of age. Among the three groups, the LC group had the highest weight at 14, 21, and 28 days of age (p<0.05), while the CON group had the lowest weight. The average weekly weight gain (AWG) was calculated based on the weekly weight data (Figure 1B). The AWG of the LC group (p<0.001) and the HC group (p<0.01) were significantly above control levels from 7-day-old to 14-day-old, but the LC group (p<0.05) was significantly lowest among three groups from 14-day-old to 21-day-old.

### Amplicon sequence variant analysis of the ileal flora

**Figure 2:**
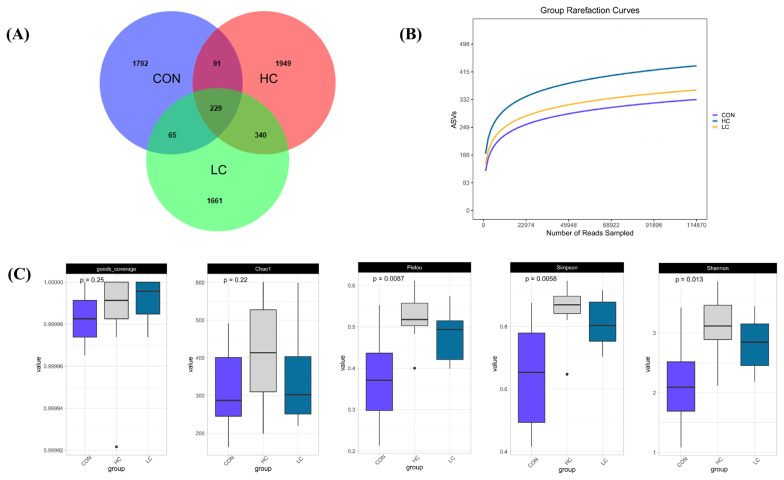
Basic analysis and alpha diversity analysis of the ileum microbiota. (A) The number of amplicon sequence variants (ASVs) that were unique to each group and shared among the three groups. (B) Rarefaction curve. (C) Alpha diversity indices.

A total of 5,701,395 high-quality sequences were obtained from all 30 samples (10 of the 30 squabs in each group) by sequencing the bacterial 16S rRNA V3+V4 region. The reads were clustered into ASV as indicated in the Venn diagram. The CON, LC, and HC groups contained 2,177, 2,295, and 2,609 ASVs, respectively (Figure 2A). There were 229 overlapping ASVs among the three groups was 229.

### Microbial richness and diversity

**Figure 3:**
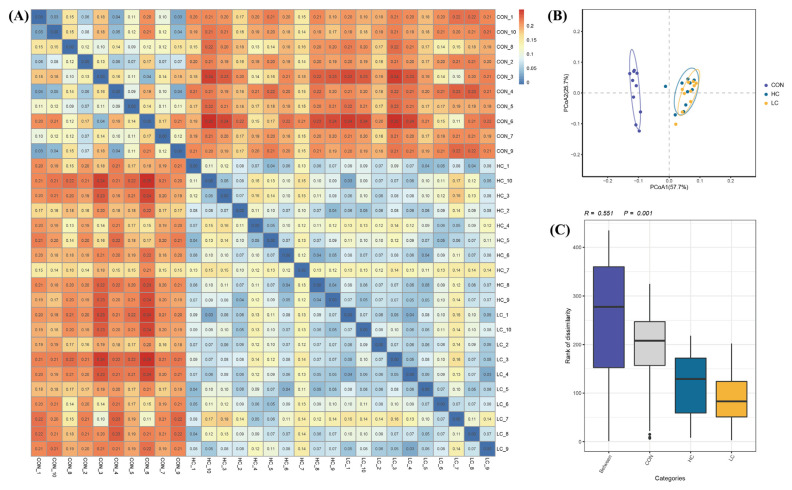
Beta diversity analysis of the ileum microbiota. (A) Heatmap based on weighted UniFrac distance analysis. (B) Principal coordinate analysis (PCoA) plots based on weighted UniFrac distance analysis. (C) Analysis of similarities (ANOSIM).

The rarefaction curve showed that the sequencing depth of the intestinal microflora in each sample was fully captured, indicating that all the samples were suitable for further analysis (Figure 2B). Alpha diversity could reflect the abundance and diversity of microbial communities in locally homogeneous habitats, as indicated by a series of statistical analysis indices (Figure 2C). No marked differences were noted in the Chao 1 index (p>0.05) or Good’s coverage index (p>0.05) among the three groups. In contrast, the corresponding Shannon (p<0.05), Simpson (p<0.01), and Pielou (p<0.01) indices significantly differed. Beta diversity analysis revealed distinct species diversity among the samples. A heatmap of the sample distance matrix, shows the hierarchical clustering of sample-to-sample distances. The β-diversity heatmap shows that the LC group and HC group have a higher similarity, and they show a separate trend compared with the CON group (Figure 3A). PCoA was used to determine the differences among the samples according to the matrix of beta diversity distance. No confidence ellipse overlap was observed between the control group and the HC or the LC group. (Figure 3B). ANOSIM confirmed marked differences among the three groups (ANOSIM: *r* = 0.551, p = 0.001) (Figure 3C). The composition of the intestinal flora changed after FMT, indicating that FMT effectively regulated the flora structure in the squab.

### Analysis of the intestinal flora structure

**Figure 4:**
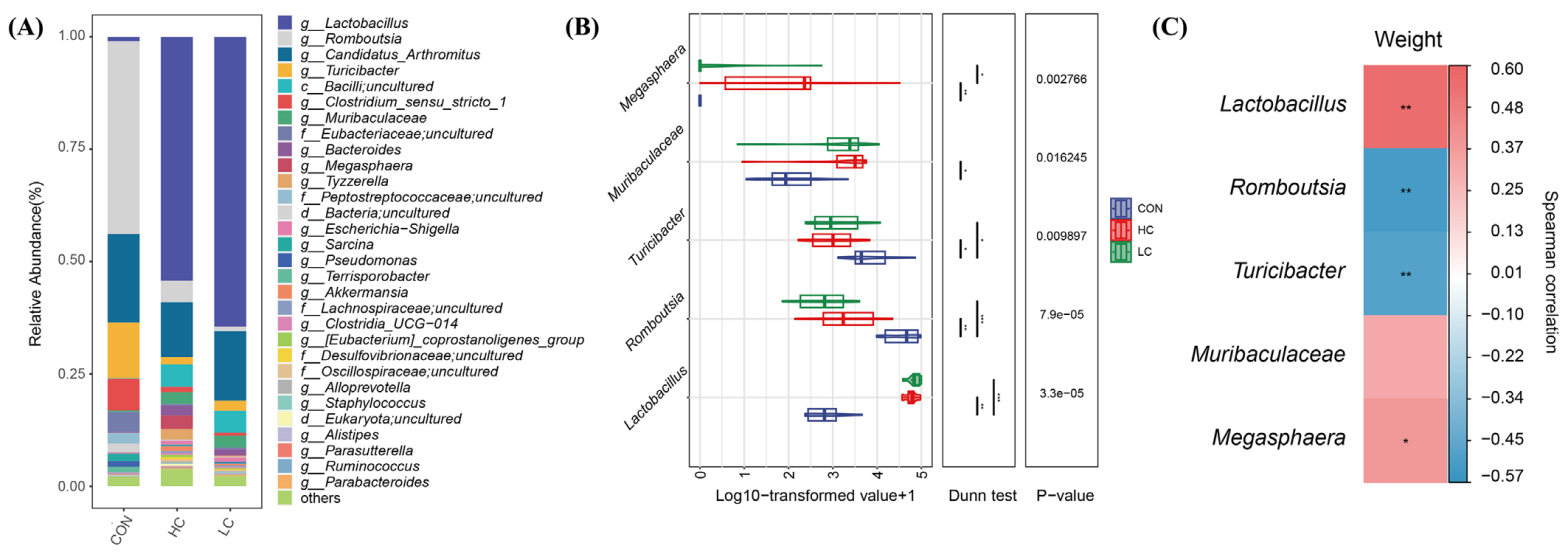
The structure of the ileum microbiota. (A) The bacterial community compositions at genus level. A relative abundance of the microbiota above 1% is listed. (B) Statistical test analysis. The box plot shows the abundance of different species in each group. Dunn test: the post hoc test. Indicates a difference at * p<0.05, ** at p<0.01, *** at p<0.001. (C) Heatmap of the Spearman’s correlation coefficient between body weight before slaughter (BWS) and five differential ileum microbiota measurements of squabs. Red and blue represents positive and negative correlations, respectively. Indicates a difference at * p<0.05, ** at p<0.01.

To further assess the effect of FMT on the intestinal flora, the intestinal microbiota composition of each group was analyzed at the genus level. Figure 4A shows the relative abundance of the major bacterial community at the genus level (only those with a relative abundance above 1% are listed). In the LC and HC groups, *Lactobacillus* (with abundance of 64.5% and 54.3%, respectively), *Candidatus_Arthromitus* (with abundance of 15.4% and 12.1%, respectively), *Bacilli, uncultured* (with abundance of 4.9% and 5.0%, respectively) were the dominant bacteria respectively. These bacteria were followed by *Muribaculaceae* (with abundance of 2.6% and 2.7%, respectively), *Turicibacter* (with abundance of 2.3% and 1.7%, respectively), *Romboutsia* (with abundance of 1.0% and 4.8%, respectively) and *Megasphaera* (with abundance of <0.1% and 3.0%, respectively). In the CON group, *Romboutsia* (43.0%), *Candidatus_Arthromitus* (19.7%), *Turicibacter* (12.4%), *Clostridium_sensu_stricto_1* (7.1%), *Eubacteriaceae*, and *uncultured* (4.5%) were the most abundant genera.

### Analysis of different intestinal flora

Five gut microflorae with differential high relative abundances were obtained after comparison of relative abundance data among the three groups using the Dunn test, including *Lactobacillus* (LC = 64.5%, HC = 54.3%, CON = 0.9%; p< 0.001), *Muribaculaceae* (LC = 2.6%, HC = 2.7%, CON = 0.3%; p<0.05), *Megasphaera* (LC<0.1%, HC = 3.0%, CON<0.1%; p<0.01), *Romboutsia* (LC = 1.0%, HC = 4.8%, CON = 43.0%; p<0.001), and *Turicibacter* (LC = 2.3%, HC = 1.7%, CON = 12.4%; p<0.01) (Figure 4B).

### Correlation analysis between intestinal flora and body weight

Spearman correlation analysis was used to analyze the correlation between body weight before slaughter (BWS) and differential gut microbiota (Figure 4C). *Lactobacillus* (*r* = 0.56, p<0.01) and *Megasphaera* (*r* = 0.38, p<0.05) were significantly positively correlated with BWS. Conversely, BWS was negatively correlated with *Romboutsia* (*r* = −0.52, p<0.01) and *Turicibacter* (*r* = −0.49, p<0.01). *Muribaculaceae* was positively correlated with BWS, but no significant correlation was detected (*r* = 0.32, p>0.05).

### Functional potential prediction of the intestinal microbiota

**Figure 5:**
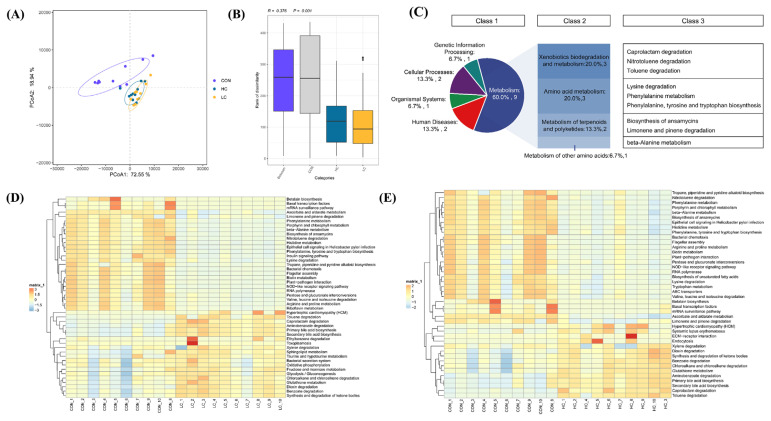
Prediction of the biological function of the ileum microbiota. (A) Principal coordinate analysis (PCoA) plots based on weighted UniFrac distance analysis. (B) Analysis of similarities (ANOSIM). (C) Statistics of 15 differential functions based on metagenomeSeq. Class 1, Class 2 and Class 3 indicate the class one, two and three Kyoto encyclopedia of genes and genomes (KEGG) pathways, respectively. (D–E) Statistical analysis of the data obtained via metagenomeSeq. Orange/yellow indicates a positive correlation. However, blue designates a negative correlation.

To further investigate the effect of FMT on the flora, we predicted and analyzed the functions of the flora in the different groups. The PCoA showed no confidence ellipse overlap of the bacterial biological function profile among the CON group and the FMT-treated groups (Figure 5A). Overall, the treatment samples were more reproducible than the CON samples were. ANOSIM among the three groups revealed a significant difference in intestinal microbial function among the CON, LC, and HC groups (ANOSIM: *r* = 0.376, p = 0.001) (Figure 5B). The results of the statistical analysis based on metagenomeSeq are visualized as a heatmap (Figure 5D–5E). Similarly, compared with those in the CON group, 35 and 16 differential biological functions of intestinal flora were detected in the LC and HC groups, respectively (*padj*<0.05). A total of 15 functions were common to all the analyses, most of which nine played a role in the metabolism Kyoto encyclopedia of genes and genomes (KEGG) pathway, namely, beta-alanine metabolism, biosynthesis of ansamycins, caprolactam degradation, limonene and pinene degradation, lysine degradation, nitrotoluene degradation, phenylalanine metabolism, phenylalanine, tyrosine and tryptophan biosynthesis, and toluene degradation (Figure 5C).

### Functional localization of the differentially expressed intestinal flora

**Figure 6:**
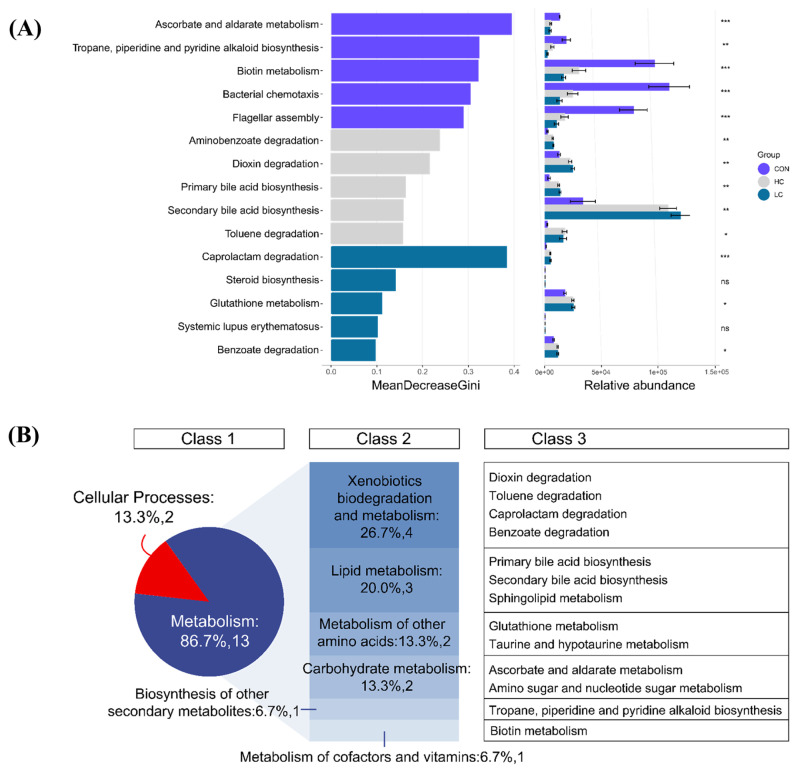
Analysis of functional differences in the intestinal flora of the CON group, HC group and LC group. (A) Statistical analysis of the random forest results. (B) Statistics of 15 differentially abundant metabolites based on random forest analysis. Class 1, Class 2 and Class 3 indicate class one, two and three Kyoto encyclopedia of genes and genomes (KEGG) pathways, respectively. CON group, control group; HC group, high-concentration group; LC group, low-concentration group. Indicates a difference at * p<0.05, ** p<0.01, *** p<0.001, ns p>0.05.

Random forest analysis was used to identify a total of 47 differential functions, and their importance was assessed. Top five differentially expressed functions were ranked by importance based on a random forest model (MeanDecreaseGini values of all the different species were >0.1) (Figure 6A). In the CON group, the top five of functional differences of the gut microbiota were the ascorbate and aldarate metabolism (*padj*<0.001), tropane, piperidine and pyridine alkaloid biosynthesis (*padj*<0.01), biotin metabolism (*padj*<0.001), bacterial chemotaxis (*padj*<0.01), flagellar assembly (*padj*<0.01). In the LC group, the top five functions related to ileal microbial differences were caprolactam degradation (*padj*<0.001), glutathione metabolism (*padj*<0.05), benzoate degradation (*padj*<0.05), sphingolipid metabolism (*padj*<0.05), and taurine and hypotaurine metabolism (*padj*<0.05). In the HC group, the top five pathways associated with functional differences in the gut microbiota were the degradation (*padj* <0.01), dioxin degradation (*padj*<0.01), primary bile acid biosynthesis (*padj*<0.01), lipid metabolism (*padj*<0.01), xenobiotics biodegradation and metabolism (*padj*<0.05). A total of 13 of the 15 functional differences in the gut microbiota among the three groups were associated with metabolism in class one KEGG pathways (86.7%) (Figure 6B). Further analysis of class two KEGG pathways revealed four significant functional differences in the gut microbiota enriched in xenobiotics biodegradation and metabolism pathway, followed by lipid metabolism pathway, which included three functional differences in the gut microbiota.

### Analyses of the correlation of functional differences with intestinal flora and body weight

**Figure 7:**
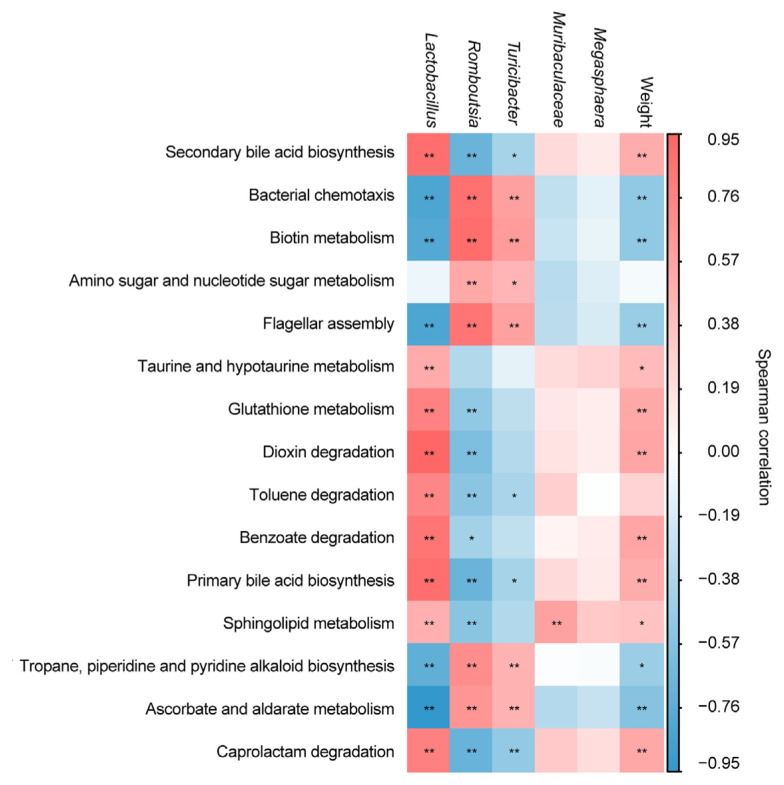
Heatmap of Spearman’s correlation coefficient between body weight, differential ileum microbiota and differentially abundant metabolites. Red and blue represent positive and negative correlation, respectively. Indicates a difference at * p<0.05, ** p<0.01.

After further analysis, we analyzed the correlations between 15 differential metabolites, five differentially abundant metabolites, and body weight of squabs through Spearman’s correlation analysis. The results are presented in Figure 7, where the left columns show that primary bile acid biosynthesis (*r* = 0.88, p<0.001), secondary bile acid biosynthesis (*r* = 0.88, p<0.001), benzoate degradation (*r* = 0.85, p<0.001), dioxin degradation (*r* = 0.95, p<0.001), ascorbate and aldarate metabolism (*r* = −0.95, p<0.001), bacterial chemotaxis (*r* = −0.82, p<0.001), flagellar assembly (*r* = −0.82, p<0.001) correlated strongly with *Lactobacillus*. Biotin metabolism (*r* = 0.90, p<0.001), bacterial chemotaxis (*r* = 0.88, p<0.001), flagellar assembly (*r* = 0.85, p<0.001) were strongly correlated with *Romboutsia*. *Turicibacter* was correlated with ascorbate and aldarate metabolism (*r* = 0.47, p<0.01), primary bile acid biosynthesis (*r* = −0.40, p<0.05), secondary bile acid biosynthesis (*r* = −0.40, p<0.05), amino sugar and nucleotide sugar metabolism (*r* = 0.45, p<0.05), toluene degradation (*r* = −0.39, p<0.05), biotin metabolism (*r* = 0.61, p<0.001), caprolactam degradation (*r* = −0.49, p<0.01), tropane, piperidine and pyridine alkaloid biosynthesis (*r* = 0.47, p<0.01), bacterial chemotaxis (*r* = 0.59, p<0.001), and flagellar assembly (*r* = 0.58, p<0.001). A statistically significant correlation was detected between *Muribaculaceae* and sphingolipid metabolism (*r* = 0.90, p<0.001). However, in all the comparisons, the differences were not statistically significant between *Megasphaera* and any of the 15 differentially abundant metabolites (p>0.05). The results of Spearman’s correlational analysis between differentially abundant metabolites and body weight are presented in the last column in Figure 7. Ascorbate and aldarate metabolism (*r* = −0.56, p<0.001), primary bile acid biosynthesis (*r* = 0.51, p<0.001), secondary bile acid biosynthesis (*r* = 0.51, p<0.001), taurine and hypotaurine metabolism (*r* = 0.42, p<0.001), glutathione metabolism (*r* = 0.54, p<0.05), sphingolipid metabolism (*r* = 0.36, p<0.001), biotin metabolism (*r* = −0.50, p<0.001), caprolactam degradation (*r* = 0.54, p<0.05), tropane, piperidine and pyridine alkaloid biosynthesis (*r* = −0.46, p<0.05), bacterial chemotaxis (*r* = −0.51, p<0.001), and flagellar assembly (*r* = −0.47, p<0.001) correlated with body weight. Amino sugar and nucleotide sugar metabolism (*r* = −0.05, p>0.05) and toluene degradation (*r* = 0.27, p>0.05) were hardly correlated with body weight.

## DISCUSSION

During the breeding and selling of squabs, body weight serves as the primary quality inspection standard, because it is closely linked to the postnatal growth and development of the squabs [17]. Extensive research has demonstrated that the gut microbiota plays a crucial role in animal growth and development [18], and FMT can effectively regulate animal growth and development [19]. The administration of FMT to adult chicken cecal microbiota has been found to enhance the body weight and feed conversion efficiency of chicks [20]. The efficacy of FMT is contingent upon the specific usage strategy employed [21]. Taken together, the findings of the present investigation indicated that squabs inoculated with the low concentration of fecal bacteria yielded more favorable weight gain than those inoculated with the high concentration. It is postulated that this outcome may be attributed to the elevated concentration of fecal bacteria, which could cause damage to the intestinal mucosal tissue [22]. Additionally, as an altricial bird, pigeons are likely to experience a critical window period for gut microbiota colonization during the first week after hatching [23]. In this study, FMT was administered during the first week after the squab hatched, and the period of the greatest weight gain in the control group occurred during the third week. Consequently, it is hypothesized that the intestinal flora of Mimas squab will reach a stable state during the third week under normal circumstances and that the associated intestinal functions are essentially fully developed. Subsequent experiments could consider dynamic tracking measurements to assess changes in the intestinal flora of squabs during FMT.

The utilization of the FMT method has been demonstrated to be a useful approach for altering the composition of the gut microbiota, thereby offering guidance for the advancement of efficacious production techniques based on the gut microbiota. Additionally, the transplantation of fecal bacteria from chickens with high feed utilization efficiency has been shown to increase the abundance of *Lactobacillus*, *Lactococcus*, and *Bifidobacterium* in the intestines of chicks [24]. The present study demonstrated a statistically significant increase in the proportion of *Lactobacillus*, *Megasphaera*, and *Muribaculaceae* in the ileum of the squabs in the FMT groups compared to that in the CON group. Conversely, the proportions of *Romboutsia* and *Turicibacter* were significantly lower in the FMT groups than in the CON group. *Lactobacillus*, a commonly used probiotic, is known to produce organic acids (e.g., lactic acid, formic acid and acetic acid) that can modulate the pH and exert antibacterial effects [25]. *Muribaculaceae* has been identified as an anti-inflammatory bacterium [26]. *Megasphaera* is usually considered a beneficial bacterium [27]; for instance, *Megasphaera elsdenii* can be administered as a probiotic to mitigate ruminal acidosis in the bovine rumen [28]. The aforementioned bacterial genera are associated with the synthesis of short chain fatty acids (SCFAs) [29,30]. Additionally, *Lactobacillus* species can potentially enhance the metabolism of SCFAs [31]. The process of gut microbiota fermentation to produce SCFAs involves the utilization of dietary fiber that remains undigested or is not easily digested by the host. This results in an increase in the host’s metabolic energy and a significant improvement in the efficiency of host feed utilization. The observed weight gain in squabs may be partially attributed to the production of SCFAs. *Romboutsia* is an inflammation-related microbe [32,33], that constituted the largest proportion of the control group. The bacterium *Turicibacter*, which is positively correlated with the levels of proinflammatory cytokines in a mouse model, is commonly considered as a pathogenic bacterium [34,35]. However, the possible function of the intestinal flora and its potential impact on animal intestinal tissue are not presently clear and explicit. In future research, we intend to analyze both the intestinal flora and intestinal tissue using whole transcriptome RNA sequencing technology. Moreover, these collaborative analyses revealed favorable correlations among *Lactobacillus*, *Megasphaera* and *Muribaculaceae* and the weight of the squab, indicating the potential for further research on this probiotic for use in squabs. However, a limited number of investigations on probiotics for domestic pigeons have tended to emphasize the influence of probiotics on pigeon immune performance and disease resistance [15,16], with minimal contributions to enhancing meat production performance in meat pigeons.

The structure of the gut microbiota plays a crucial role in determining its function, which in turn interacts with the host’s gut function to jointly determine the host’s physiological and health status [36]. Through functional prediction and analysis, this study identified 15 core differential functions of the gut microbiota, 86.7% of which were related to metabolism, which aligns with the findings of previous research [24]. Additionally, this study conducted a joint analysis and determined the robust positive correlation between primary and secondary bile acid biosynthesis and the differential abundance of the bacterium *Lactobacillus* in terms of core differential functions. Prior research has demonstrated the involvement of *Lactobacillus* in the metabolism of BAs, whereby *Lactobacillus* catalyze the conversion of primary bile acids into secondary bile acids (SBAs) through the production of hydrolytic enzymes known as bile salt hydrolases [37,38]. The production of SBAs by *Lactobacillus* is of considerable importance in the maintenance of intestinal health in animals. The inclusion of deoxycholic acid, derived from secondary bile acids, in the diet of chickens can mitigate the inflammation of the ileum caused by *Clostridium perfringens* infection in a dose responsive manner [39]. Moreover, the findings of this investigation indicate that the variance in squab weight can also be attributed to the xenobiotic’s biodegradation and metabolism, too. The microbiota plays a crucial role in the metabolism of heterobiotics within the intestinal tract, thereby contributing to the maintenance of a stable intestinal environment [40]. Nevertheless, the joint analysis findings revealed that certain differential functions, such as carbohydrate metabolism, exhibit minimal contributions to the variation in the final weight of squabs, and even display negative correlations. This outcome could be attributed to the reference database utilized in the functional prediction process. Currently, there is a dearth of academic inquiry into the squab, with a greater emphasis on referencing other species for functional prediction. This may result in potential discrepancies in the prediction of intestinal flora function in squabs. Further research is warranted to advance the understanding of the specific mechanism underlying the impact of FMT on squab weight.

## CONCLUSION

In summary, administering FMT could considerably augment the weight of squabs. Importantly, at the time of gavage, the optimal dose was 200 μL of bacterial solution with a concentration of 0.1 g/15 mL. Additionally, in comparison to those in the control group, the fecal bacteria transplantation enhanced the abundance of advantageous bacteria such as *Lactobacillus* and *Megasphaera* in the intestines of squabs but led to a decrease in the abundance of pathogenic bacteria such as *Romboutsia* and *Turicibacter*. Alterations in the intestinal microbiota composition of squabs have resulted in notable modifications in the metabolic functions associated with intestinal flora lipids, amino acids, heteropoietic factors and other related factors. These changes may account for the excellent growth performance observed in squabs induced by FMT. Consequently, FMT is an effective strategy for enhancing the body weight of squabs.

## Figures and Tables

**Table 1:** Composition and nutrient levels of pellet diet and raw grain in basic diet (air-dry basis)

Items	Value
Pellet diet^1)^	
Metabolizable energy (MJ/kg)	11.50
Crude protein (%)	20.00
Ether extract (%)	12.00
Calcium (%)	0.98
Total phosphorous (%)	0.59
Raw grain	
Ingredient (%)	
Corn grain	46.7
Sorghum grain	14.6
Wheat grain	17.3
Peas	21.4
Total	100.0
Calculated nutrients^2)^	
Metabolizable energy (MJ/kg)	12.60
Crude protein (%)	12.59
Ether extract (%)	2.83
Calcium (%)	0.08
Total phosphorous (%)	0.32
Available phosphorus (%)	0.12

1) The pellet diet was purchased from Henan Aoke Biotechnology Co., Ltd (Luoyang, Henan, China).

2) Nutrition values were calculated from tables of feed composition and nutritive values in China (31st edition, 2020).
